# Lake Sediment Records on Climate Change and Human Activities in the Xingyun Lake Catchment, SW China

**DOI:** 10.1371/journal.pone.0102167

**Published:** 2014-07-17

**Authors:** Wenxiang Zhang, Qingzhong Ming, Zhengtao Shi, Guangjie Chen, Jie Niu, Guoliang Lei, Fengqin Chang, Hucai Zhang

**Affiliations:** 1 Key Laboratory of the Plateau Surface Process and Environment Changes of Yunnan Province, Key Laboratory of Plateau Lake Ecology and Global Change, Yunnan Normal University, Kunming, China; 2 Key Laboratory of Humid Subtropical Eco-geographical Process, Ministry of education, Fuzhou, China; Agharkar Research Institute, India

## Abstract

Sediments from Xinyun Lake in central Yunnan, southwest China, provide a record of environmental history since the Holocene. With the application of multi-proxy indicators (total organic carbon (TOC), total nitrogen (TN), δ^13^C and δ^15^N isotopes, C/N ratio, grain size, magnetic susceptibility (MS) and CaCO_3_ content), as well as accelerator mass spectrometry (AMS) ^14^C datings, four major climatic stages during the Holocene have been identified in Xingyun′s catchment. A marked increase in lacustrine palaeoproductivity occurred from 11.06 to 9.98 cal. ka BP, which likely resulted from an enhanced Asian southwest monsoon and warm-humid climate. Between 9.98 and 5.93 cal. ka BP, a gradually increased lake level might have reached the optimum water depth, causing a marked decline in coverage by aquatic plants and lake productivity of the lake. This was caused by strong Asian southwest monsoon, and coincided with the global Holocene Optimum. During the period of 5.60–1.35 cal. ka BP, it resulted in a warm and dry climate at this stage, which is comparable to the aridification of India during the mid- and late Holocene. The intensifying human activity and land-use in the lake catchment since the early Tang Dynasty (∼1.35 cal. ka BP) were associated with the ancient Dian culture within Xingyun’s catchment. The extensive deforestation and development of agriculture in the lake catchment caused heavy soil loss. Our study clearly shows that long-term human activities and land-use change have strongly impacted the evolution of the lake environment and therefore modulated the sediment records of the regional climate in central Yunnan for more than one thousand years.

## Introduction

The Yunnan Plateau, southwest China, is located in the confluence zone of the Asian monsoon, and the climate is mainly controlled by a system comprising the Asian southwest monsoon, westerly winds and local climatic influences of the Qinghai-Tibet Plateau. Since the Cenozoic, a large number of structurally-controlled lake basins formed following the uplift of the Qinghai-Tibet Plateau [1]. Therefore, it is a key area in which to study the prehistoric Asian monsoon patterns over different time scales.

The climatic transition from the Pleistocene to Holocene changed the activity characteristics and life style of ancient people [2]. Agriculture provided the material foundation for birth of civilization and promoted an increase in population and intensity of human activity [3], [4], which has had a lasting and profound impact on the environment. China is the origin and development centers of rain-fed millet [5] and cultivated rice [6]. Early wheat remains in China emerged mainly in the Tarim Basin [7], the Hexi Corridor [8], [9], the Tianshui Basin [10], Shandong and Henan [11], dated at roughly in late Holocene, and the cultivated rice spread southwardly to Southeast Asia through Guangdong and Yunnan Provinces [12] at the same time.

Lake sediments play an increasingly important role in the research of global change and regional environmental evolution because they can demonstrate continuity and environmental and seasonal sensitivity, therefore providing high resolution and typically abundant environmental and climatic information [13], [14]. Whilst both natural climate change and human-influenced environmental changes are likely to be recorded in Holocene sediments of Yunnan [1], it is critical to be able to evaluate the influence of human activities on the reliability of sedimentary proxies in inferring the past climate change [15], [16]. Few researchers have investigated climate change and potentially associated human-influenced environmental change based on lake-catchment sediments, at millennial time scales.

Based on high-resolution geochemical proxy analysis of sediment core from Xingyun Lake, we aim to establish the processes and stages of the environmental change of Xingyun′s catchment, and identify the initial time and manner in which human activities influenced the receiving environment and additionally probe how Xingyun Lake responded to the interaction between climate and humans.

## Study area

**Figure 1:**
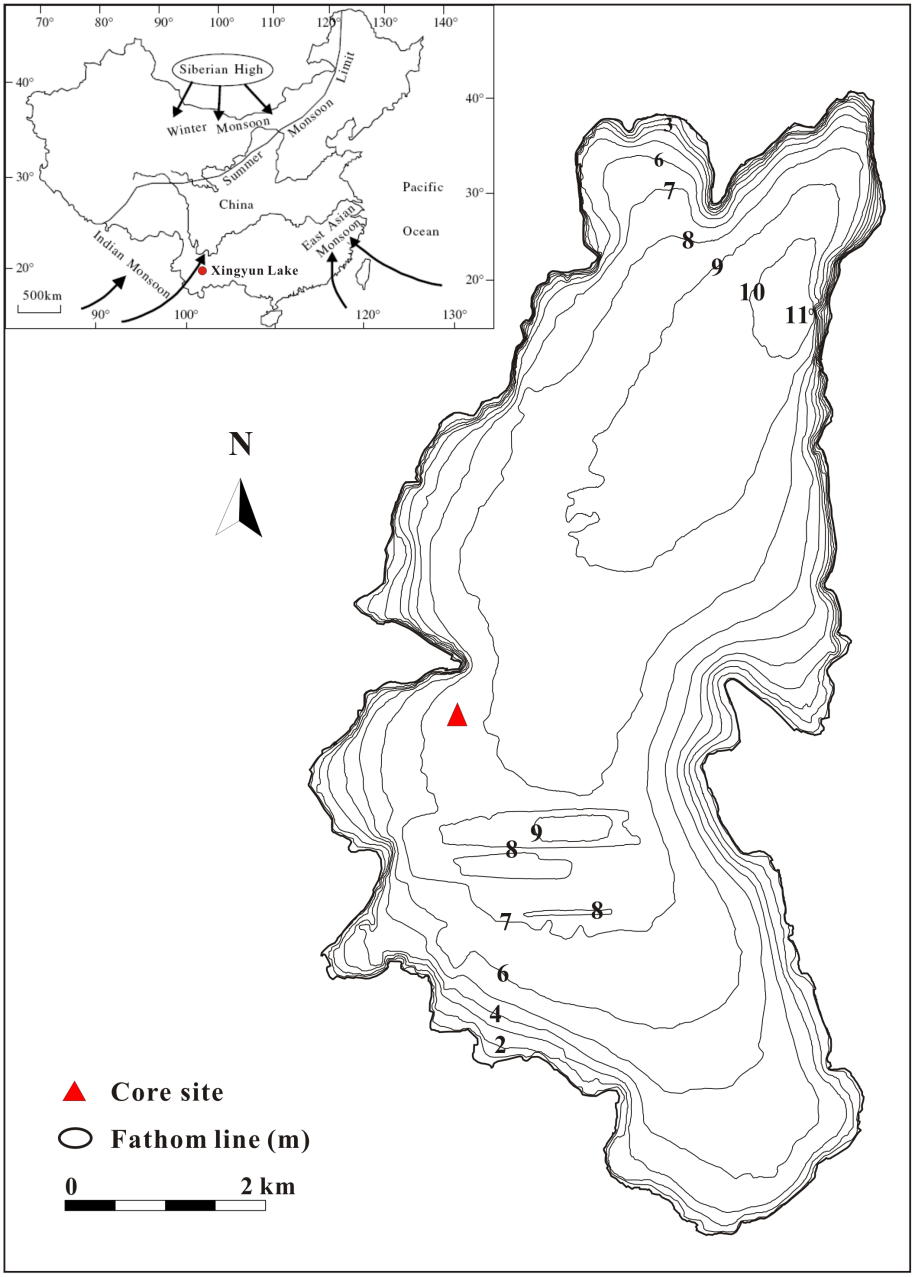
The monsoonal system in China and the location of Xingyun Lake (Based on Wu [19]).

Xingyun Lake is located in the central Yunnan Plateau (Figure 1), about 80 km south of Kunming and 20 km east of Yuxi. It is a semi-closed shallow lake, situated at 1740 m above sea-level and is surrounded by mountains. The lake has a water surface area of 34.7 km^2^ and catchment area of 325 km^2^. The mean water depth is approximately 7 m. The lake joins Fuxian Lake to the north via the narrow channel of the Gehe River [17]. Mean annual temperatures recorded at regional weather stations are in the range of 12–16°C, and the mean annual precipitation is approximately 976 mm, with more than 85% falling between May and October [18]. The climate in the catchment is dominated by the Asian southwest monsoon. The native vegetation comprises mainly broadleaved deciduous forests [16]. The small catchment area of Xingyun Lake suggests that the lake sediments will likely be reliable recorders of environmental change of the catchment, given the limited external influence thereon.

## Materials and Methods

In June 2011, a sediment core 429 cm in length was recovered using a piston corer at the western part of the deep basin of Xingyun Lake in water 7.5 m deep (24°19′48.78″ N; 102°45′ 47.04″ E, Figure 1). No specific permissions were required for the described study, which complied with all relevant regulations. The field studies did not involve endangered or protected species and specific location can be seen below. The core was sampled in the field at 2.0 cm intervals, and sub-samples were sealed in plastic bags for transport to the laboratory.

The experimental analysis of sedimentary proxies was carried out at the Key Laboratory of Plateau Surface Process and Environment Changes of Yunnan Province. Magnetic susceptibility was measured with a Bartington MS2 magnetic susceptibility meter and mass-specific magnetic susceptibility (χ_lf_) was also calculated. The CaCO_3_ content of the samples was measured using the calcimeter method of Bascomb [20]. This involved measuring the amount of CO_2_ produced after adding the HCl, and stoichiometrically calculated this into CaCO_3_ content, and error of <1% was achieved during this analysis. Grain size was measured with a Mastersizer-2000 laser diffraction particle size analyzer (Malvern Instruments Ltd., UK) after treatment with H_2_O_2_ and HCl to remove organic matter and carbonate [21]. From this analysis, the relative standard deviation of parallel analyses for individual samples obtained was <1%. In the interest of obtaining accurate data, all of above proxies were measured three times, under the same conditions, treatment and analytical methods.

Total organic carbon (TOC), total nitrogen (TN), δ^13^C and δ^15^N isotope ratios of the samples were measured at 4.0 cm intervals at the Key Laboratory of China Geological Survey of Nanjing Center, Geological institution of the Ministry of Land and Resources, using an elemental analyzer (Flash EA1112 HT, Thermo) and mass spectrometer (MAT253). Samples were decarbonizes with 0.5 mol/L HCl and then rinsed with deionized water until the filtrate was neutral, and then freeze dried. Afterwards, samples were weighed into tinfoil capsules and combusted at 1080°C in excess oxygen. The resulting material was flushed into the MAT253 with a He-carrier flow for analysis [22]. Isotope ratios are reported in δ-notation, where δ = (R_s_/R_st_−1)×1000. R_s_ and R_st_ are the isotope ratios of the sample and the standard (PDB for carbon, AIR for nitrogen), respectively. Standard sample (lake sediment of the national soil standard reference material, GSS-9) with known δ^13^C and δ^15^N were measured daily to monitor analytical accuracy. The analytical precision was better than the mean: ±0.1‰ for δ^13^C, ±0.2 ‰ for δ^15^N, 0.1 mg·g^−1^ for TOC and 0.05 mg·g^−1^ for TN.

## Results

### 1 Age model and chronology

**Figure 2:**
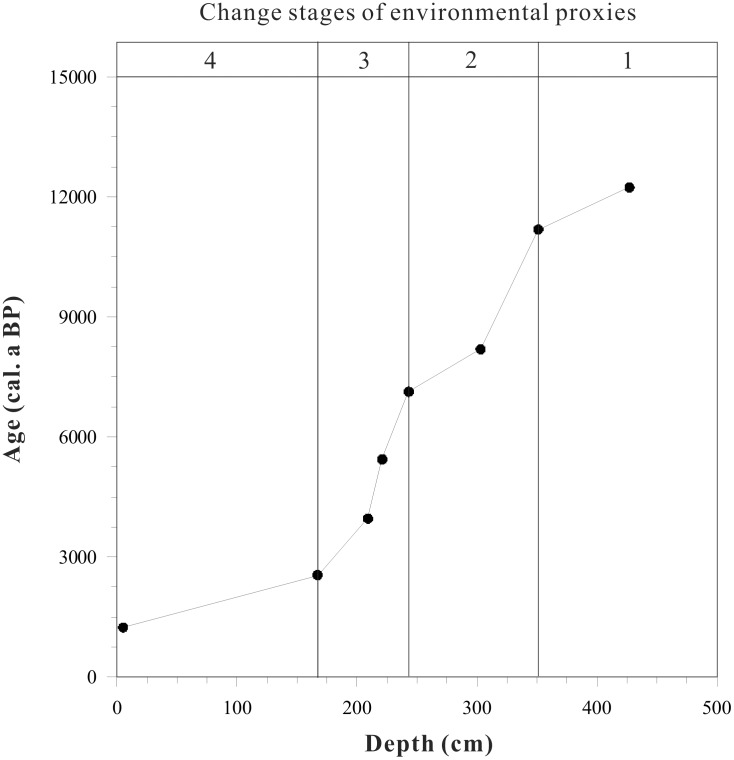
Age-depth curve and sedimentation rate for Xingyun lake core. Radiocarbon dates are listed in Table 1.

Eight bulk sediment and plant macrofossil samples were collected from the organic-rich horizons of the core, and accelerated mass spectrometry (AMS)^ 14^C dates were measured at the AMS Laboratory of Beijing University, China (Table 1 and Figure 2). Organic carbon was extracted from each sample and dated following the method described by Nakamura [23]. The conventional ages were converted to calibrate with IntCal13 calibration data [24]. Based on linear-fitting analysis, the uppermost sediments in the core yield an age of 1.2 ka. According to ^210^Pb dating of the core [16], the anomalously old age can be considered to result from “carbon reservoir effects” on radiocarbon dating of the Xingyun Lake sediments. To produce the age model for the core, we subtracted the reservoir age of 1.2 ka from all the ages, assuming that it is constant throughout the core (Table 1). The age models and the records of environmental proxies show a continuous sedimentation record, extending back to the last ∼11 ka BP. The sedimentation rate of the upper layer (0–167 cm) is 1.25 mm/a and 1.24 mm/a, and is 0.17 mm/a and 0.27 mm/a in the lower layers (167–243 cm and 243–351 cm), respectively. The sedimentation rate in the lowest part of the core (351–429 cm) is 0.72 mm/a (Table 1). The results of sedimentation rate are in coincidence with geochronology of Xingyun Lake and other lakes in Yunnan Province [13], [25].

**Table 1:** Radiocarbon dating in Xingyun Lake sediment core and age model.

Laboratory number	Depth (cm)	Dating material	δ^13^C	AMS ^14^C age (^14^C yr BP)	Calibrated ^14^C (2σ, cal a BP)	Median (cal a BP)	Modelled age (cal a BP)	Sedimentation rate (mm/a)
PA06849	5	Organic matter	−31.42	1335±40	1310–1175	1240	40	1.25
PA06850	167	Organic matter	−26.20	2485±40	2725–2365	2550	1350	1.24
PA06851	209	Organic matter	−19.92	3625±40	4080–3840	3960	2760	0.30
PA06852	221	Organic matter	−24.02	4645±40	5570–5305	5440	4240	0.08
PA06853	243	Organic matter	−26.46	6210±40	7250–7005	7130	5930	0.13
PA06854	303	Organic matter	−22.21	7375±40	8325–8050	8190	6990	0.57
PA06855	351	Organic matter	−28.20	9760±45	11250–11110	11180	9980	0.16
PA06856	427	Organic matter	−30.84	10360±45	12405–12050	12230	11030	0.72

### 2 Proxy indices

**Figure 3:**
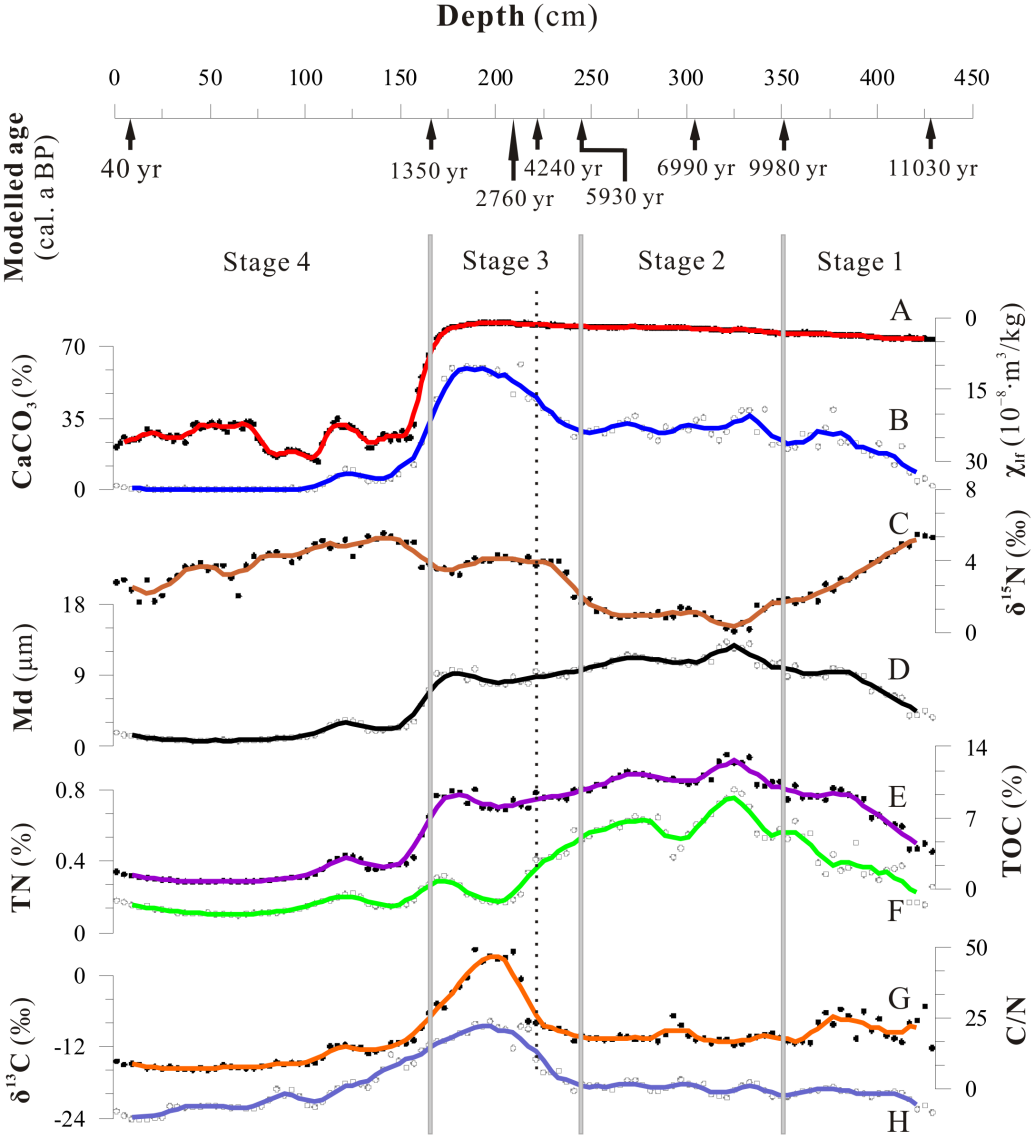
Multi-proxies results from Xingyun lake sediments: (A) magnetic susceptibility χ_lf_ and CaCO_3;_ (B) grain size and δ^15^N; (C) TOC and TN; (D) C/N and δ^13^C. ^14^C AMS control points for Xingyun core are denoted on upper axis, and each curve is the 5 data running average value of the proxies. The gray bars highlight the boundary between Stage 1(429–351 cm, 11060–9980 yr BP, Younger Dryas and the early Holocene), Stage 2 (351–243 cm, 9,980–5930 yr BP, strongest summer monsoon in the Holocene), Stage 3 (243–167 cm, 5930–1350 yr BP, weaker summer monsoon) and Stage 4 (167–0 cm, 1350 yr BP to present, intensified human activities).

The χ_lf_ value of Xingyun Lake sediments varies from 0.9 to 30.1×10^−8^ m^3^/kg with an average value of 10.9×10^−8^ m^3^/kg, and demonstrates a substantial increase above 167 cm in the core (Figure 3-A). An average value of the χ_lf_ from 167 cm to the top is 24.24×10^−8^ m^3^/kg. The CaCO_3_ content of Xingyun Lake samples is in the range of 0–61.45% (Figure 3-B), and is hence variable and fluctuates substantially. In the 243–167 cm interval, CaCO_3_ content peaks at 61.5% (213 cm), and is very low (average of 0.32%) from 109 cm to the top of the core. The medium diameter (Md) is in the range of 2.88–15.11 µm, with an average of 6.63 µm. The sediment is therefore composed principally of fine grain sizes, with the finest material located between 167 cm and the top of the sediment core (Figure 3-D).

**Figure 4:**
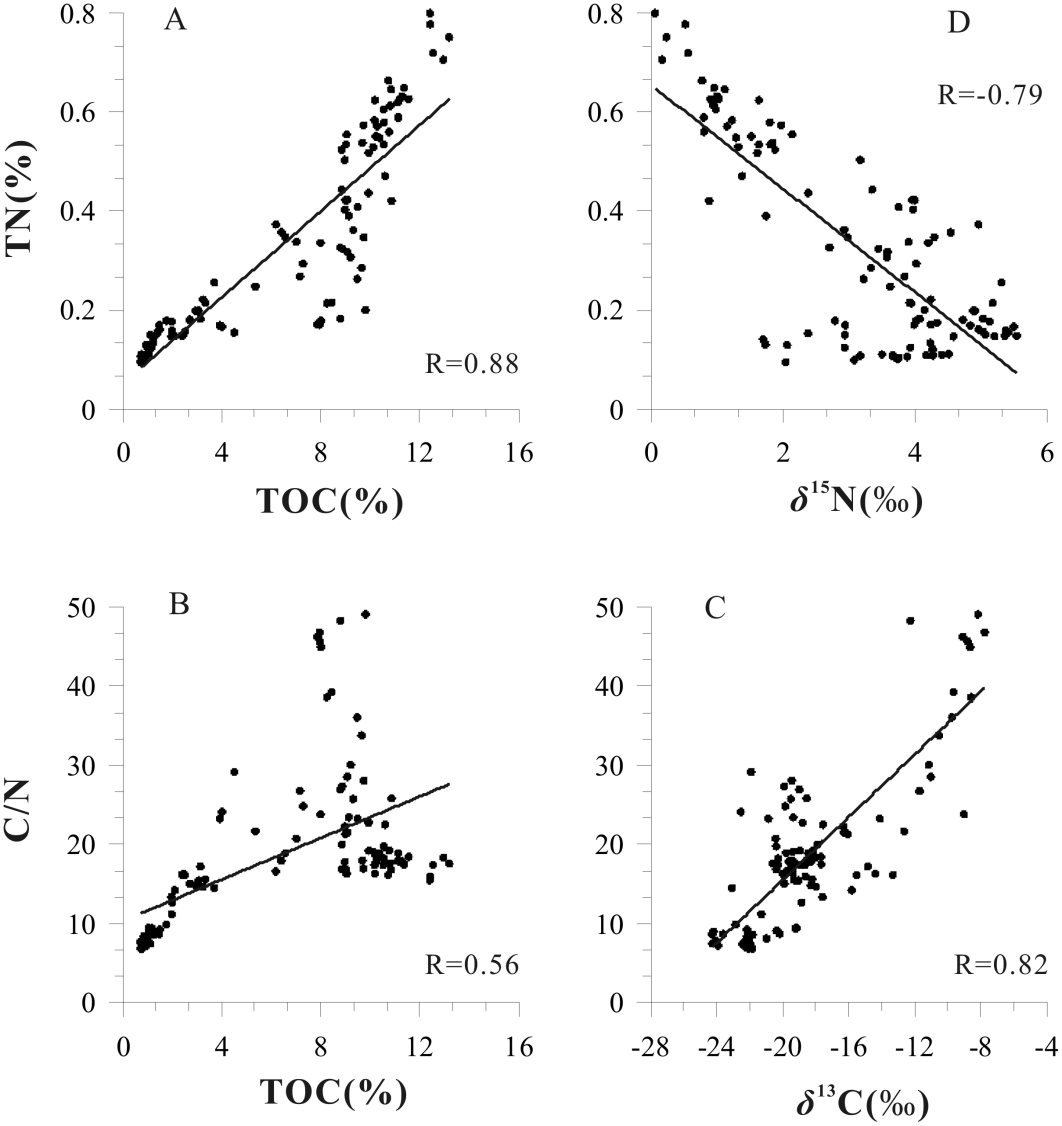
Correlations between TOC contents, TN contents, C/N ratios, δ^13^C and δ^15^N (n = 108). The correlations were all highly significant (P<0.01).

Values of TOC vary between 0.73% and 13.20% with a mean value of 6.40% (Figure 3-E), and gradually increase between 429 cm and 351 cm. The TOC content increases to a peak value from 351 cm to 243 cm although there are obvious fluctuations. The lowest value of TOC (approximately 0–3%), from 167 cm to the top of the sediment core, indicates a decrease in the abundance of plants in the catchment. The TN content is generally low, with an average of 0.33% (Figure 3-F), and is well correlated with TOC (R = 0.88, Figure 4-A). The C/N ratio of the sediments varied between 6.88 and 49.07, with an average of 18.71 (Figure 3-G). The C/N ratio exceeds a value of 20 at the 429–161 cm interval of the sediment core, with the maximum value between 237 cm and 161 cm. From 109 cm to the top of the core, the C/N ratio is ≤10. The C/N ratio displays a positive correlation with the TOC (R = 0.56, Figure 4-B), and a very weak correlation with the TN content (R = 0.11). This suggests that the TOC more strongly influences the C/N ratio.

Values of δ^13^C vary from −24.27‰ to −7.74‰ with an average value of −18.37‰ (Figure 3-H). The highest values of δ^13^C were found in the 243–167 cm interval of the sediment core. There is a demonstrable positive correlation between δ^13^C and the C/N ratio (R = 0.82, Figure 4-C). Values of δ^15^N vary between 0.07‰ and 5.55‰ with an average value of 3.11 (Figure 3-C), and the 351–243 cm interval of the sediment core is the most ^15^N depleted (minimum of 0.07‰). The depth profile of δ^15^N is similar to that of TN content, and there is a good inverse correlation between δ^15^N and TN content (R = −0.79, Figure 4-D).

## Discussion

### 1 Climatic significance of the proxy indices

#### Geochemical proxy indices

Sources of TOC in lacustrine sediments have been suggested to be the biomass of aquatic algae, aquatic macrophytes and terrestrial plants in the lake catchment. The contribution of each of these different organic matter types to the TOC is affected by the regional climates, catchment environment and human activity. For example, there would be little terrestrial plant contribution to the lake sediments if deforestation of wooded areas occurred [26], [27]. The TN content of lake sediments is typically indicative of lake’s trophic level, and is closely related to the algae production [28].

The C/N ratio is regarded as an effective indicator of the provenance of organic matter in sediments [29], [30]. When sedimentary organic matter primarily originates from endogenous materials, it has a C/N ratio between 4 and 10 because of protein-rich and cellulous-poor aquatic organisms. Exogenous materials tend to result in C/N ratios greater than 20 owing to the protein-poor and cellulous-rich nature of higher terrestrial plants [31], [32]. Lamb [33] suggested that terrestrial plant matter always result in C/N ratios of between 16 and 20 in lacustrine sediments.

Variations in δ^13^C are closely related to the sources of organic matter in the lake sediments [34]; C3 and C4 land plants have δ^13^C values that range from –37‰ to –24‰ and –19‰ to –9‰ with an average value of –27‰ and –14‰, respectively [35]. Crassulacean acid metabolism (CAM) plants have a broad range of δ^13^C values and the δ^13^C composition of aquatic macrophytes is complex and broad of range. The δ^13^C values of aquatic plants vary between −20‰ and −12‰. These plants take up carbon from HCO_3_^–^ in lake water for photosynthesis, yielding higher δ^13^C values than for emergent plants (−30‰ to −24‰).

The δ^15^N values of lake sediments can be influenced by the concentration of dissolved nitrate, nitrogen-fixing processes, bacterial decomposition, kinetic isotopic effects and climate change [36]. The dominant factor influencing δ^15^N depends on the local physicochemical processes [37]. In general, large amounts of terrigenous organic matter entering the lake would cause a low δ^15^N value during warm-humid period and vice versa [32], [38]. Moreover, human activities can increase the δ^15^N value in lacustrine sediment significantly [39].

#### Other proxy indices

In southwest China, especially in Yunnan Province, rainfall is a grain-size determining factor. In short time-scale and high resolution studies (years to decades), it is evident that larger sediment grain-sizes become more migration during high rainfall periods in a wet climate [40]. Sediment grain size can, therefore be used to study the changes in humidity and can be distinguished from environmental indicators linked to the grain size of aeolian sediments. Moreover, the development of agricultural practices is expected to compromise the soil surface structure, and therefore increase the content of finer sediments being transported and entering lake [41].

Magnetic susceptibility is an efficient and sensitive proxy with which to study the lake environment [42]. The research results indicate that ferromagnetic material, concentrated in surface soil and detritus of the lake catchment [43], is one of the main sources of magnetic minerals in the lake sediments. CaCO_3_ in the lake sediments is mainly composed of authigenic and allochthonic carbonates. The two main factors that induce carbonate precipitation are biological, and physico-chemical, such as temperature variations, evaporation and release of CO_2_ [44].

### 2 Environmental evolution of Xingyun Lake

**Figure 5:**
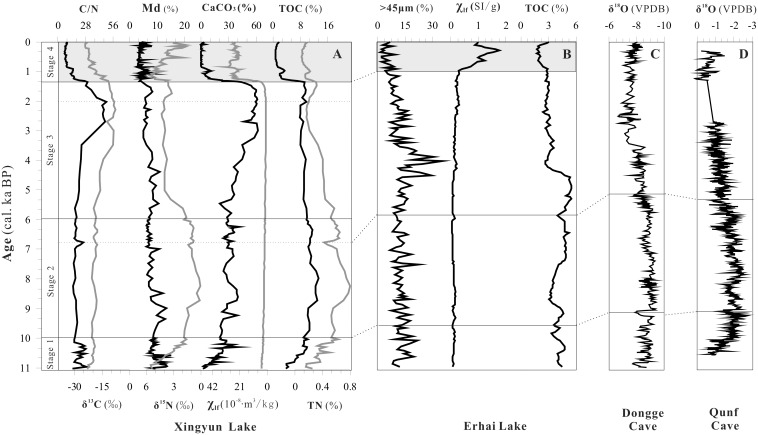
Comparison of the climatic records from Xingyun Lake with the climatic proxies of Erhai Lake and the δ^18^O of Dongge Cave and Qunf Cave in the Holocene. The period of intensive human activities and the farming agriculture of Xingyun and Erhai Lake was denoted by the gray area. (A) The climatic proxies of Xingyun Lake indicating strength of Asian summer monsoon and record the strengthened human activities in Yunnan plateau; (B)The climatic proxies (grain size >45 µm, χ_lf_ and TOC content) of Erhai Lake [1]; (C) The stalagmite δ^18^O record from Dongge Cave, southwest China [45]; (D) The stalagmite δ^18^O record from Qunf Cave, Oman [51].

From the environmental proxies and depositional rates determined for the Xingyun lake sediments, the environmental evolution, including monsoonal and human influences, can be divided into four main stages (Figures 3 and 5), as follows:

#### Stage 1∶11.06–9.98 cal. ka BP (429–351 cm)

This period was characterized by a gradual increase in TOC, TN content, CaCO_3_, χ_lf_ and fluctuating Md, indicating a period of high productivity, enhanced hydraulic conditions and increasing precipitation in the lake catchment. The C/N ratio, δ^13^C and relatively low δ^15^N value indicate that organic matter in the lacustrine sediments was mainly derived from C4 land plants and that the climate was warm and humid. The change in environmental proxies are considered consistent with an intense influence of the Asian southwest monsoon and overall the climate shifted from cold-wet to warm-humid during this early Holocene period [45].

#### Stage 2∶9.98–5.93 cal. ka BP (351–243 cm)

Compared with the early Holocene, this period experienced abundant rainfall and high effective moisture of the lake catchment, as represented by the high values of Md, CaCO_3_ and χ_lf_. The high TOC and TN content, stable C/N and δ^13^C values suggest that the organic matter was mainly derived from land and aquatic plants, and that vegetation in the lake catchment was flourishing. The low δ^15^N value is consistent with a climate characterized by warm and wet conditions strongly influenced by the southwest Asian monsoon. During this period, the warmest stage identified lies in the 8.98–6.10 cal. ka BP interval, which coincides with the global Holocene Optimum [46]–[50].

#### Stage 3∶5.93–1.35 cal. ka BP (243–167 cm)

Unit 1 5.93–4.36 cal. ka BP (243–217 cm) Between 5.93 and 4.36 cal. ka BP, the high TOC, TN content, C/N ratio, the positive δ^13^C value and gradually increasing δ^15^N value indicate significant climatic fluctuation and a decrease in plant productivity in the lake catchment. This is consistent with the relatively high CaCO_3_ content, overall fine grain size and lower χ_lf_. The environmental proxies therefore denote the beginning of a temperature decrease, consistent with a climatic cold event reported from other localities in southwest China in the 6–4 ka BP interval [13]. However, there is no immediate prospect of human activities records of Xingyun Lake sediment in this period.

Unit 2 5.60–1.35 cal. ka BP (217–167 cm) The transition from Unit 1 to 2 is marked by a strong shift in environmental proxies. The highest CaCO_3_ content and the lowest χ_lf_ show that the temperature was comparatively warm, and the high TOC, C/N ratio, low TN content, positive δ^13^C and high δ^15^N values all suggest that organic matter entering the lake at this time was mainly derived from C4 terrestrial plants and planktonic productivity was low. The environmental proxies, and relatively slow rate of deposition, are further consistent an ecological change, decreased precipitation and intensification of evaporation within the lake catchment. The climate appeared to warm and dry at this stage, comparable to the warm and dry period of the mid- to late Holocene [51] and aridification of India [52]. Cultural relics unearthed from the Lijiashan ruins in this period depicted a deer falling prey to two leopards [53], indicating that the climate of the Xingyun catchment was warmer than presently. Meanwhile, the massive people immigrated into Yunnan Province, which coincided with the Spring and Autumn and the Warring States Period (∼2.0 ka BP) of Chinese history, and it had further exacerbated the ecological environment of the lake catchment.

#### Stage 4∶1.35 cal. ka BP to present (167–0 cm)

The consistently low content of CaCO_3,_ TOC and TN reflect the low biomass of the Xingyun catchment during this stage. The fine grain-size, high rate of deposition and high χ_lf_ suggest a heavy loss of soil from the catchment. The C/N ratio, δ^13^C and δ^15^N values are consistent with high contributions of organic matter from aquatic plant life to the lake sediments, and negligible terrestrial organic matter input. This suggests an abrupt change in the plant community, and also that intensified human activity and land-use in the catchment began from 1.35 cal. ka BP. This is consistent with the period of Dian culture established within the Xingyun catchment [54]. At this time extensive deforestation and development of agriculture in the lake catchment caused heavy loss of soil and fine-grained sediment was easily transported into the lake. In addition, the intensive agriculture would likely have led to organic matter decomposition and release of bound soil particles – as demonstrated by the increase in magnetic minerals present in the lake sediments. During the early Tang Dynasty (600 AD), local population increased in the Xingyun area, which was related to a high rate of immigration into Yunnan. This area was developed into a social and economic center of stockbreeding, irrigation, agriculture and trade [55]. Increased human activity, such as deforestation, reclaiming of land, agriculture and stockbreeding, resulted in the reduced vegetative coverage of the landscape. This inevitably led to increased erosion of surface soil and destruction of the vegetation ecology, as demonstrated by the high χ_lf_, low TOC and TN content, and increased δ^15^N value as a result of human activity.

### 3 Comparison with Erhai Lake, Qunf and Dongge Cave

To better understand the regional environment change of Xingyun lake catchment, the records of the lake environment are compared to those with high temporal resolution records from different monsoonal regions. Erhai lake is lied on the area of southwest monsoon, which is about 300 km distant from Xingyun lake. Qunf cave (54°18 E, 17°10 N) is located in Oman experienced typical Indian monsoon climate [54], and Dongge cave (108°5 E, 25°17 N) is situated in southwest China, with local climate influenced by both India monsoon and East Asian monsoon [47].

As shown in Figure 5, the changing trend of the environmental proxies of Xingyun Lake was similar with the TOC content of Erhai Lake and the δ^18^O value of Qunf and Dongge stalagmite. They exhibit a similar feature of the Holocene summer monsoon variation: intensive summer monsoon in the early Holocene, a decreasing trend during the middle Holocene, and a relatively weak summer monsoon in the late Holocene. Asian monsoon intensity was found to be directly controlled by mid-July solar insolation at 30°N over the entire Holocene. The general consistency of monsoon records of Xingyun Lake with those from other continental records across Asia indicates an in-phase relationship between the Asian summer monsoon on orbital timescales over the Holocene.

The Xingyun Lake records (TOC, and δ^15^N isotope, grain-size, CaCO_3_) show a distinct weakened monsoon event during 8.3–8.1 cal. ka BP, and this 8.2-ka BP weak monsoon event is also clearly exhibited in the δ^18^O records in caves of Qunf and Dongge, but to a less degree in the records of Erhai Lake. Meanwhile, the Holocene Optimum of Qunf cave was terminated in 7300 a BP, which was earlier than the lake of Xingyun Lake, Erhai and Dongge cave [1], [45], [51]. This may be due to the fact that insolation-driven Intertropical Convergence Zone migrated southward and India summer monsoon began to decrease, which resulted in less precipitation and gradual influence to north direction after 7.3 cal. ka BP.

During the late Holocene, the records of TOC, TN and CaCO_3_ in Xingyun Lake, TOC content of Erhai Lake, the δ^18^O value of Qunf and Dongge cave indicate the weak summer monsoon. The abrupt changes of χ_lf_ and fine-grained of Xingyun and Erhai Lake sediment suggest the intensive human activity and land-use since 1.35 ka BP, which wasn’t recorded in Qunf and Dongge cave. It suggests that this symbiotic human-monsoon relationship may have existed at Yunnan Plateau, southwest China. However, human activity had less influence on cave system compared to that on the lake catchment which was important for agriculture activities, and there stalagmite records can provide more faithful information on climate change.

## Conclusions

Based on the application of biochemical (TOC, TN content, δ^13^C and δ^15^N isotope ratios, C/N ratios) and other environmental proxies (grain-size, χ_lf_, CaCO_3_), as well as AMS ^14^C dating and historical documents, we have identified four climatic stages in the Holocene as recorded in lacustrine sediments on Xingyun Lake. Furthermore, we have identified the impact of human activities during the past ∼2000 years on the environmental records. The conclusions of this study can be summarized as follows:

(1) A δ^15^N isotope record from a 429-cm sediment core from Xingyun Lake, dating back to 11.06 cal. ka BP, provides the first complete Holocene nitrogen isotope record for this area. The record reveals a long history of climate changes and human influence in the lake catchment in the latter part of this history. The Holocene climatic evolution is characterized of a shift from being dry-cold to humid-warm and finally to dry-warm. There is also evidence that the lake expansion resulted from an intensification of the Asian southwest monsoon during the early Holocene.

(2) Between 9.98 and 5.93 cal. ka BP, the gradually expanded lake may have reached the optimum water depth, causing a marked decline in the coverage of aquatic plants and low planktonic productivity in the lake, again strongly influenced by the Asian southwest monsoon. This coincided with the global Holocene Optimum. Between 5.60 and 1.35 cal. ka BP the temperature was comparatively warm and precipitation decreased, thus comparable to the aridification of India in the mid- and late Holocene. Meanwhile, the massive people immigrated into Yunnan Province, which coincided with the Spring and Autumn and the Warring States Period (∼2.0 ka BP) of Chinese history, and it had further exacerbated the environment of the lake catchment.

(3) Human activity and land-use intensified since the early Tang Dynasty (∼1.35 cal. ka BP) are corresponded well with the development of the local Dian culture. The extensive deforestation within the catchment could lead to heavy soil loss as revealed in lake sediments.
